# Catalytic hydrocracking of jatropha oil over natural clay for bio-jet fuel production

**DOI:** 10.1038/s41598-023-40500-2

**Published:** 2023-08-17

**Authors:** S. H. Hassan, N. K. Attia, G. I. El Diwani, Sh. K. Amin, R. S. Ettouney, M. A. El-Rifai

**Affiliations:** 1 Chemical Engineering and Pilot Plant Department, National Research Center, Dokki, Cairo, Egypt; 2 Chemical Engineering Department, Faculty of Engineering, Cairo University, Giza, Egypt

**Keywords:** Chemical engineering, Climate-change mitigation

## Abstract

Currently, the conversion of biomass to produce high-valued biofuels such as biodiesel and bio-jet fuel has attached booming interests, when used for partial replacement of petroleum fuels in different ratios is a promising solution due to the problem of depleting petroleum reserves and environmental purposes. Non-edible Jatropha oil can be transformed to biofuel when subjected to were hydrocracking at hydrogen pressure using an activated natural clay as a catalyst in a high pressure batch reactor. The type of product and its quality and quantity depend on the process conditions such as reaction time, temperature, and catalyst type, form, and amount. The present work aims to study the hydrocracking process of Jatropha oil at different operating conditions. The catalyst is characterized using SEM, FTIR, XRF, and XRD. The effect of process conditions variation have been studied and discussed. The results showed the highest yield of 40% bio-jet fuel was achieved at a temperature of 350 °C, H_2_ pressure of 4 bar, and reaction time of 18 min. the bio-jet fuel products were tested and their specifications were conformed to ASTM D1655 specifications, viz the freezing point (−56 °C), the flash point (53 °C), and existent gum content (5.9 mg/100 ml).

## Introduction

Several challenges face the demand and supply of energy in the world. The increase in petroleum fuel consumption worldwide affects fossil fuel reserves. The daily global total consumption of petroleum reached 99.56 million barrels in 2022. The estimated petroleum oil reserves will be drained in less than fifty years, at the current consumption rate of 2.7% annually ^1,2^. Also, the emissions that are exhausted from fossil fuels combustion are large contributors to global warming and environmental pollution^3–5^. Environmental awareness, depletion of fossil fuels and the increase in energy consumption and price‏ are the main factors leading to the search for alternative energy resources to substitute fossil‏ fuels^4,6–8^.

Renewable energy sources decrease the effect of greenhouse gases and are superior to fossil fuels with respect to their lower SO_x_, CO_,_ and CO_2_ emissions^9^. Representative renewable energy source technologies include fuel‏ cells, hydropower, solar power, geothermal energy, wind power, biofuel, and hydrogen production^10,11^.

Biojetfuel is‏ one of the most important sources of renewable and green energy expected to gradually replace fossil fuels in the near future with increased blending ratios. It is anticipated to reach 25% in 2020, 30% in 2030, and 50% in 2050^12–15^. Biofuels can be produced from several agricultural raw materials, through different production methods depending on the required final products and feedstock^16^. These are mainly vegetable oil based and biomass. Vegetable oil-based feedstock includes edible and non-edible oils, waste cooking oil, jatropha, jojoba, rapeseed, castor and microalgal oil. Biomass feedstock includes waste materials, aquatic biomass, energy crops, and forest products ^9,10,14,17^.

Jatropha curcas seems to be the best plant to use in the production of second-generation biofuels, which are fuels made from non-food crop feedstock and agricultural or forestry waste^18^. J. curcas can be grown on unproductive and marginal areas. Since J. curcas grows on degraded soils and doesn't compete with agricultural output as a non-food feedstock, its cultivation for biodiesel production doesn't result in a change in land use. It can be planted in desert or along farmers field boundaries, the cost of plantation is largely incurred in the first year and improved planting material can make a huge difference in yield, raising jatropha plant and its maintenance create a jobs. The annual productivity of jatropha seeds in Egypt about 1.36 ton/feed in 1st year and 3.4 ton/feed in 3rd year irrigated with 6000 m^3^/fed/year municipal waste water primary treated^19^. A byproduct of oil extraction, such as seedcake, can be composted to create organic fertilizer, which can then be utilized as organic manure^20^. This could lower N_2_O emissions brought on by fertilizers containing nitrogen. Last but not least, J. curcas may present a chance for developing nations to profit from the rising demand for biofuels^21^. The plant Jatropha curcas L. is robust, pest and drought resistant, and inedible to animals. It is mostly grown in tropical nations as a hedge to shield agriculture from cattle, sheep, and goats.

Biofuel production processes range between catalytic cracking, pyrolysis, transesterification, and fermentation^18^. Using the proper catalyst, hydrocracking is one of the best routes to produce biofuels from oils. This is due to many reasons firstly, the catalytic hydrocracking operating temperature range of (350–450 °C) is lower than pyrolysis temperatures of (500–850 °C), also the choice of feedstock used in pyrolysis plays a significant role in the quality of the final product. Secondly, the reaction time of the catalytic hydrocracking process is much shorter than that required in the fermentation process. Production of ethanol via fermentation necessitates pretreatment processes such saccharification and hydrolysis. Finally, catalytic hydrocracking can produce several fractions of petroleum cuts such as gasoline, kerosene, diesel, and mazot compared to the transesterification method that produces only biodiesel^19–21^.

Bio-kerosene or bio-jet fuel, used mainly in air transportation, is one type of biofuel having the same characteristics of jet-fuel according to ASTM D1655 and contains the same components. There are two types of jet-fuel: civil aviation (Jet A, Jet A-1, Jet B) and military jet fuels “(JP.1- JP.10, JPTS, Zip fuel, Syntroleum)^9,13,14^. These are produced by different processes based on the feedstock which could either be a gas, sugar, alcohol, or oil^22^. The catalytic hydrocracking process is considered the best choice to convert vegetable oil to bio-jet fuel in one step^15^.

The catalytic hydrocracking of “non-edible and edible oils” demands the development of the appropriate cracking catalysts and the selection of the proper reactors for the production of bio-jet fuel^23–25^ . Pore size, acidity, and shape all affect the catalyst's characteristics. Various catalyst types exist, including macroporous (silica-alumina and alumina), mesoporous (MCM-41 and SBA-15), microporous (zeolite), and composite microporous-mesoporous materials. ^10,19,26^, Ni/ZSM-5 ^27^, Ni–Mo/Al_2_O_3_^28^, CoMo/Al2O3, Ni-HZSM-5/SBA-15(Weng et al., 2015),and Ni–W/SiO_2_–Al_2_O_3_, PdNi/HZSM-5 catalyst ^29^, K-modified zeolite ZSM-5^30^, NiMo/SA-2 and NiMo/Z-10, CoMo/MCM-41(Ooi et al.), Mesoporous-Alumina-Supported CoMo Catalysts^31^, Ni/SAPO-11(Zuo et al.),Ni/H-Beta^28^, and ZSM-5-Zn^32^.

The catalytic reaction process in heterogeneous catalysis proceeds as follows^33^: Surface reactant diffusion, adsorption inside the catalyst surface, surface reactant diffusion of products, products desorption from the surface, and surface reactant diffusion away from the surface^34^.

The aim of this work is the production of bio-jet fuel through hydrocracking of Jatropha oil by using prepared low cost inorganic catalysts from natural clay to decrease the price of biofuel production. In order to the objectives of economic, environmental, and also to achieve sustainable development saves sources of raw materials for future generations. Catalyst characterization will be undertaken and the reaction products will be evaluated for the modified natural clay as catalyst. Comparison between the conventional jet fuel and the produced bio-jet fuel will be undertaken by chemical and physical analyses to verify the bio-jet fuel conformity to ASTM D1655 specifications.

## Materials and methods

### Materials

- Jatropha Curcas seeds were obtained from the Ministry of Agriculture. Jatropha were successfully grown in Egypt and can be cultivated arid land and using municipal primary treated waste water, in many places in Egypt. Jatropha oil is extracted in National Research Center, Egypt.
- The used catalyst is The Egyptian Bentonite **B** which obtained from Borg El Arab, Alexandria, Egypt. It comes from Egyptian quarries and it is used in the manufacture of ceramics, pottery and handicrafts.
- Hydrochloric acid HCl was purchased from Sigma – Aldrich laborchemikalien GmbH Company, Germany for the catalyst preparation of a concentration of 37%.

### Methods

#### Extraction of Jatropha oil

Jatropha oil is extracted using hexane from crushed Jatropha seeds. The extraction was carried out in a soxhlet apparatus at 60–70 °C. The volume ratio of hexane to Jatropha seeds was 5:1. Hexane is separated and recovered from extraction mixture by vacuum distillation at 40 °C. The extracted oil yield is about 25% weight of grounded seeds. Table 1 summarizes the physical and chemical properties of Jatropha oil while Table 2 represents its fatty acids profile.

**Table 1:** Physical and chemical properties of Jatropha oil.

Viscosity 40°C (m.Pa.s)	Density (kg m^-3^)	Specific Gravity	Iodine value g I_2_/100 g oil	Peroxide value m Mol kg^-1^	Molecular Weight (g/mol)
28.8	825	0.825	23.6296	297.62	900

**Table 2:** Fatty acid composition of extracted Jatropha oil.

Fatty acid	% composition
Palmitic acid	18.22
Stearic acid	5.14
Oleic acid	28.46
Linoleic acid	48.18
Phorbol esters	0.0018

#### Catalyst preparation

The catalyst preparation and modifications that are produced and used in the catalytic hydrocracking of oil depend on many previous surveys and research to add value to the mineral earth clay use in a wide range of industrial applications^19,35,36^. The Catalyst is prepared by adding 50 gm of Bentonite clay (B) to a prepared 500 ml of 0.1M HCl and the mixture is heated to110 °C in a round flask connected to a condenser, for 4h. The resulting Bentonite clay suspension is then rapidly quenched by 500ml ice-cold distilled water. After quenching, the suspension medium was filtered and washed several times using distilled water until chloride ions Cl^-1^ are removed by the washing water. The modified Clay is then dried in an oven at 110 °C and then calcinated at 550 °C for 4h. The dry paste is then ground to the powder form.

#### Catalyst characterization

**Figure 1:**
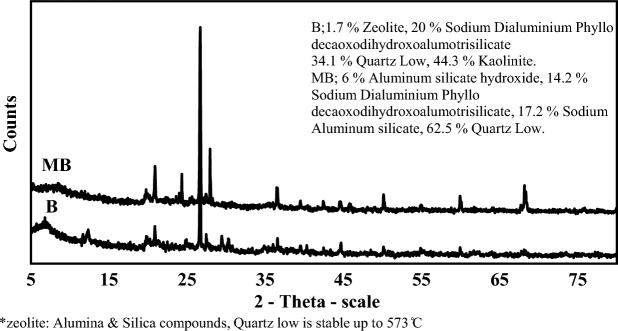
XRD pattern of raw (B) and modified catalyst (MB).

**Figure 2:**
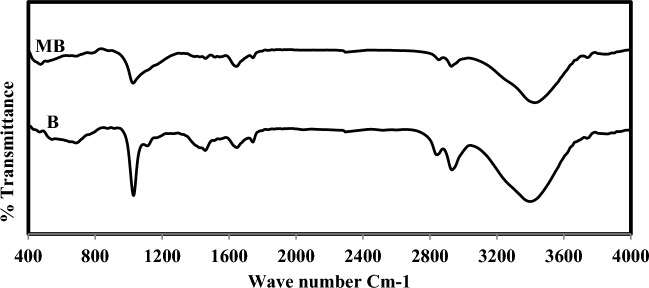
FTIR pattern of B and MB.

**Figure 3:**
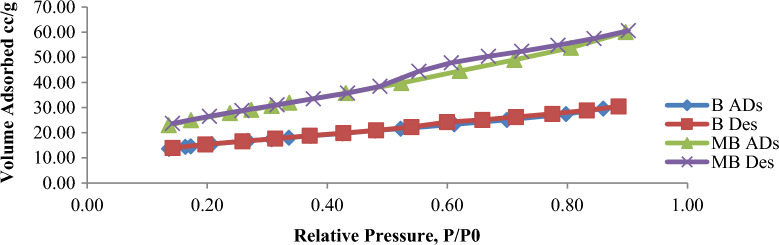
Adsorption–desorption isotherm of B and MB.

- X-ray fluorescence spectrometry (XRF) analyses were carried out on an AXIOS, panalytical 2005, Wavelength Dispersive (WD–XRF) Sequential Spectrometer. X-ray diffraction (XRD) tests to confirm the crystal structure were carried out with the following settings:" Goniometer = Theta/Theta; Minimum step size 2 Theta: 0.0001; Minimum step size Omega: 0.0001, Sample stage = Stage for flat samples/holders, Diffractometer system = EMPYREAN, and Anode Material: Cu. The scans were taken at 2θ = 4–80°.
- Fourier transforms infrared spectroscopy (FTIR) analysis was performed using Origin Jasco Infrared Spectrum (FT/IR-6100type A) over the wave number range 4000–399 cm^-1^.
- The surface area of the inorganic catalysts and raw materials were studied using version 1.21 of the Quanta Chrome TouchWin™ to determine the Brunauer, Emmett, and Teller (BET) surface area. Before the experiments all material samples were degassed at 200 °C for 6 h. The "BET" specific surface area was estimated from the linear part of the adsorption curve.
- The total pore volumes of micro and meso pores were directly obtained from nitrogen adsorption at P/PO around (0.1–0.98)". "The pore diameter distribution was calculated from the desorption branch using" "Barret Joyner Haleda, BJH formula^37–39^". It is important to ensure that the purity of the adsorptive is not less than 99.999%. In addition, the accuracy of the results depends on careful preparation and sampling of the adsorbent^40^.
- The produced catalyst specifications are shown in Tables 3 and 4, which illustrate XRF analysis and BET surface area properties. XRD pattern, Fourier transforms infrared spectroscopy; FTIR and adsorption–desorption isotherm of raw and treated catalyst are shown in Figs. 1, 2, 3 respectively.

**Table 3:** XRF analysis of raw (B) and modified catalyst (MB).

Main Constituents (wt%)	B	MB
SiO_2_	48.7	67.2
TiO_2_	1.2	1.6
Al_2_O_3_	19.19	19.75
Fe_2_O_3_tot	9.55	5.4
MnO	0.1	0.01
MgO	2.06	0.87
CaO	4.5	0.35
Na_2_O	1.37	0.57
K_2_O	1.19	1.53
P_2_O_5_	0.25	0.10
SO_3_	1.28	0.09
Cl	0.44	0.02
LOI	10.17	2.50

**Table 4:** Specifications of raw material (B) and modified Catalyst (MB).

Sample property	Molecular weight (g/mol)	Cross section area (°A^2^/mol)	Total pore volume (cm^3^/g)	S_BET_ (m^2^/g)	Average pore diameter (nm)
B	28.013	16.2	0.048352	52.2959	3.6984
MB	28.013	16.2	0.10388	96.2112	4.3186

It is obvious from XRF in Table 3 that SiO_2_ and Al_2_O_3_ are the major components of all raw materials and prepared catalysts, with trace amounts of other metal oxides. The hydrochloric acid effect on raw materials is to increase the silica content and to decrease the abundance of metal oxides such as CaO, Na_2_O, Fe_2_O_3_, MnO, Ca^2+^, Na^+^ cations. It is seen that in XRD (Fig. 2), modifications exist in the clay peaks on treatment with HCl. The peaks illustrate the intensity and width of catalyst which are nearly the same as the pure raw materials. This indicates structural preservation. The peak severity is an index of the degree of laminar structure retained by the clay^35,41^.

The Egyptian clay contain bands as seen in FTIR Fig. 1 which indicate H–O–H stretching of absorbed water, C–H stretching, and OH stretching, and hydration of clay minerals respectively. The prepared catalyst MB shows band due to H–O–H stretching of absorbed water, C–H stretching, OH stretching hydration, Si–O stretching, and Si–O–Si bending^35,41^. It is observed in Table 4 that the prepared catalysts have increased pore size and a wide range of distribution. This indicates good reactant distribution during the catalytic process. The adsorption–desorption isotherm curves of the prepared catalysts are shown in Fig. 3. It is seen that the modified catalysts have a higher capability of adsorbing the reactants on their surface and a higher products desorption from the catalyst surface.

#### Bio-jet fuel preparation

**Figure 4:**
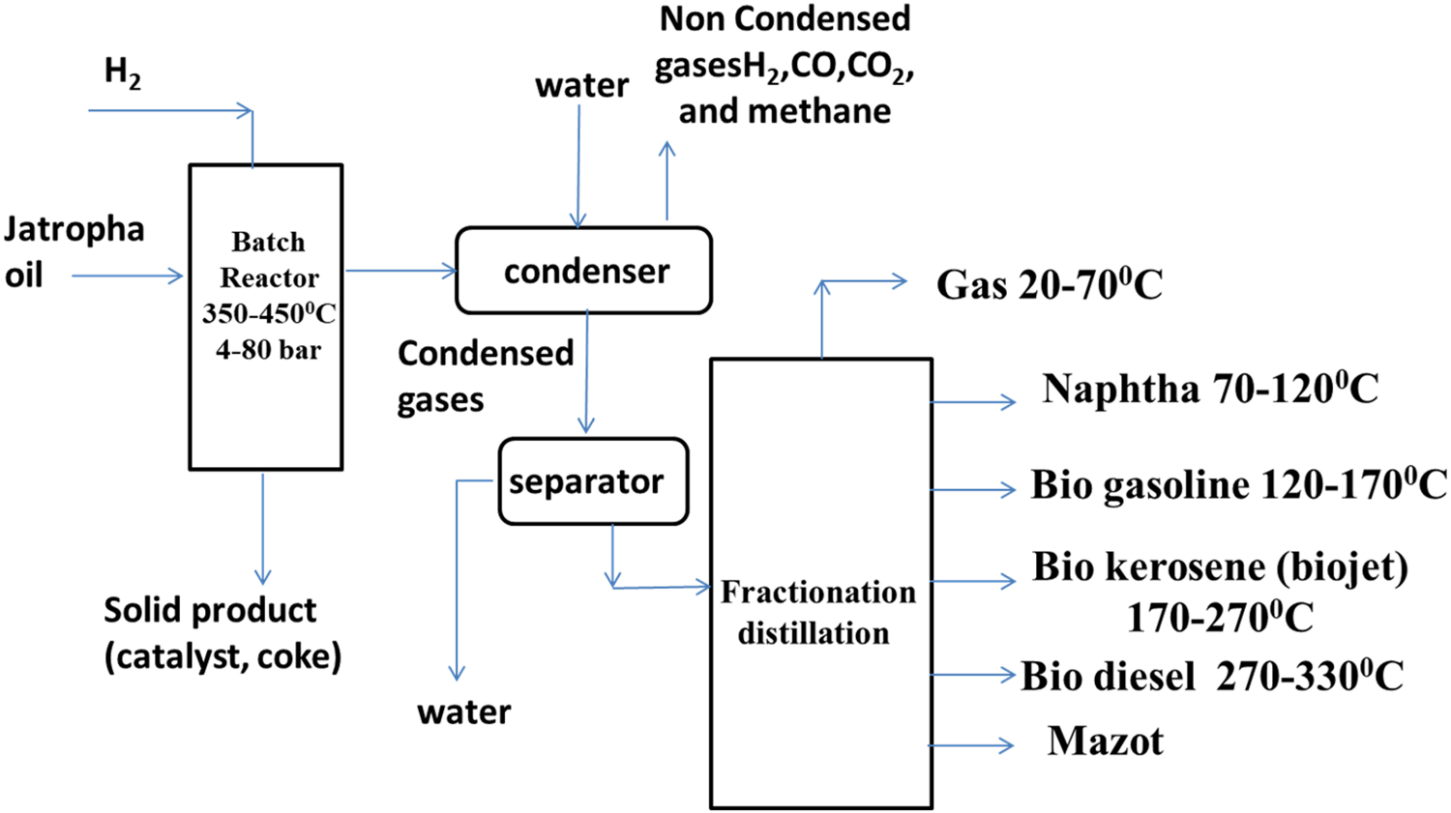
Flow sheet of bio-jet fuel production steps at optimum conditions (350 °C, 18 min, 4 H_2_ bar, and 4% MB).

**Figure 5:**
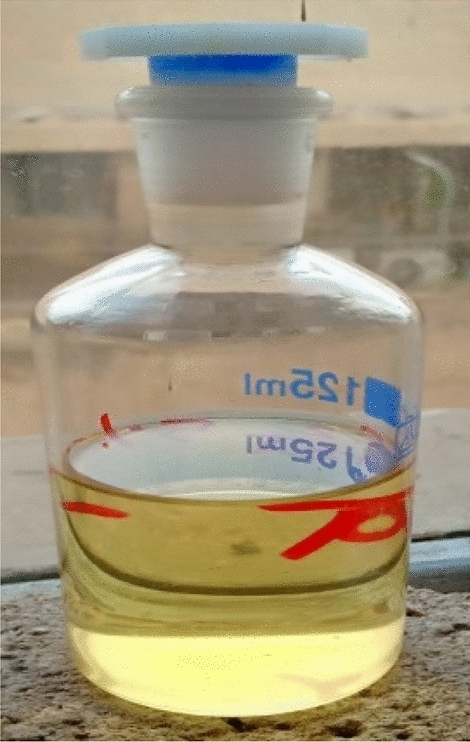
Produced bio-jet fuel at optimum reaction condition after fractional distillation (170–270 °C).

Catalytic hydrocracking was implemented as follows: 100 gm of filtered Jatropha oil is charged in a stainless-steel bench scale high pressure high temperature batch reactor (450ml). The prepared catalyst was added in different weight ratios to the Jatropha oil. Hydrogen gas is compressed into the reactor to maintain a pressure of 4 bars. The mixture was heated to reach the required temperature of 350–450 °C for a time interval of 10 to30 minutes. The collected product was then centrifuged to separate any suspended particles from the produced bio fuel mixture. The biofuel mixture was fractionated in a laboratory fractional distillation unit to obtain bio-gasoline at a temperature range of (60–170 °C), bio-jet fuel at a temperature range of (170–270 °C) and biodiesel at a temperature range of (270–330 °C), this process is presented in a simplified flow sheet which illustrated in Fig. 4. The produced bio-jet fuel after fractional distillation is presented in Fig. 5.

#### Produced bio-jet fuel characterization

The produced bio-jet fuel is characterized according to ASTM D1655 specifications. The following properties are measured.

##### GC/mass analytical procedure

Thermo Scientific’s ISQ Single Quadruple MS, TG-5 MS fused silica capillary column (30 m, 0.251 mm, 0.1 mm film thickness) was used in gas chromatography/mass spectrometry (GC/MS) to determine the composition of the product fuel. An electron ionization device with an ionization energy of 70 eV (electron volts) was employed for GC/MS detection. As a carrier gas, helium is used at a constant flow rate of 1 ml/min. The temperature of the column oven is initially maintained at 60 °C, then increased by 5 °C /min to 200 °C, kept for 2 min, and then increased to 280 °C (10 °C /min).

##### Preparation of bio-jet fuel blend engine testing

The blend of jet A-1 (kerosene) with 5% of produced bio-jet from jatropha oil was compared to that of jet A-1 after being tested in a prototype jet engine (Jet-Cat 80/120).

##### Freezing point

The freezing point of bio-jet fuel samples is measured according to ASTM D2386 specifications. 15 ml from every sample are placed in a glass tube and placed in liquid nitrogen until the sample is totally freeze. The freezing point was measured by reading the thermometer indicator point. This occurs when temperature reading stabilizes at the last thin crystal layer in the solid–liquid mixture.

##### Flash point

The flash point of bio-jet fuel or any volatile material is the lowest temperature at which fumes of the liquid will ignite in presence of a source of ignition. The flash point is a descriptive property used to distinguish flammable fuels, Petrol and combustible fuels, such as diesel. A fuel with a flash point less than 37.8 °C is called flammable, whereas fuels are called combustible when the flash point temperature is above 37.8 °C.

According to the ASTM D2887 specification, the PENSKY MARTENS FLASH POINT TESTER is used to estimate the flash point of bio-jet fuel. The sample is contained in an open / closed cup which is heated at different rates. A flame is brought over the liquid surface at sufficient height and the cup is opened with every degree increase in temperature up to the moment of vapors ignition.

##### Viscosity

Viscosity is a property used to measure the resistance of a specific fluid to flow. The sample is measured by a laboratory “DV-II + Pro” Viscometer which determines the viscosity of a specific fluid by a given shear stress and shear rates depending on the specific liquid temperature and a drive motor with different rpm.

##### Density

The density of fluids is measured using Hydrometers. It is a laboratory floating body made from a glass material which contains a cylindrical stem and a bulb loaded with a metal weight. Depending on the depth that plunges within the sample, the density value is determined directly from the reading scale at the top part of the glass body with its measuring units.

##### Existent gum content

According to ASTM D-381, the existent gum content of jet fuel must not exceed 7 mg/100 ml^12^. The test was carried out in the laboratory of Misr Petroleum Company, at Ghamra, Egypt. The gum values are estimated for the blend of 5% vol. bio-jet fuel with Jet A-1.

### Experimental research and field studies on plants

“The present study complies with the international, national or institutional guidelines” .

## Results and discussion

### Bio-jet fuel production

#### Effect of % catalyst

The bio-jet fuel process is affected by many operating conditions such as temperature, reaction time, catalyst types, the catalyst to oil ratio, etc. Tables 5 and 6 show that at the reaction temperature of the hydro catalytic cracking of 350 °C, the reaction is exothermic. For the same reaction conditions of 18 min, 350 °C, and 4 bar H_2_, different percentages of MB catalyst: 1%, 2%, 3%, 4% were added to produce bio-jet fuel. The reaction mixture temperature is elevated to about (100 ± 10 °C), the highest temperature reached was 452 °C when using 4% MB catalyst and the lowest temperature reached was 420 °C when using 1% MB catalyst.

**Table 5:** Operating conditions of catalytic hydrocracking of jatropha oil.

% Catalyst MB	Time (min)	Reaction pressure (bar)	Final reaction temperature (°C)
Effect of % catalyst			
4%	18	80	452
3%	18	70	445
2%	18	70	430
1%	18	70	420
No-catalyst	18	65	420
Effect of time			
1%	18	70	420
2%	10	40	420
2%	20	80	420
2%	30	80	435

**Table 6:** Biofuel specification and the yield of 1^st^ three fractionations from ASTM D1655 distillation.

Modified Catalyst (MB)	Density (gm/ml)	Viscosity at 40°C (m.pa.s)	% volume of product		
			Bio-gasoline 170 °C	Bio-jet 270 °C	Bio-diesel 340 °C
No-catalyst 18 min	0.879	3.75	45.5	33.8	–
4% MB, 18 min	0.878	5.15	20	40	18.7
3% MB, 18 min	0.8878	5.3	24.35	39.7	–
2% MB, 18 min	0.8864	4.9	31.16	33.76	–
1% MB, 18 min	0.867	4.6	30.3	33	–
2% MB, 10 min	0.904	10.8	22.5	18.3	–
2% MB, 20 min	0.8735	5	34.5	31	–
2% MB, 30 min	0.87	4.5	33	38	–

The constant hydrogen inlet pressure of 4 bars is used during the reaction to saturate double bonds and helps all cracking reactions to occur. The highest constant pressure reached in the batch reactor was 80 bar when using 4%MB at 18 min and the lowest pressure of 65 bar was reached in case of no catalyst is used at a the same reaction time of 18 min.

The pressure during the reaction system is an indicator of product quality and a good achievement of cracking reaction. The non-condensable gases, after cooling, that are produced from the reaction, result in an increase from 4 to 20 bars. These are carbon dioxide, carbon mono oxides, and C_1_–C_4_ aliphatic compounds.

**Figure 6:**
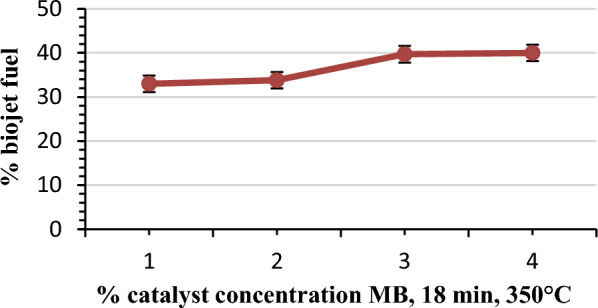
Catalyst to oil ratio effect on yield of bio-jet fuel production.

The results of these experiments are shown in Table 6 and Fig. 6. It is seen that the bio-jet fuel production yield increased with the increase of MB percentage. The 4% MB gave the best yield followed by 3% MB (40% and 39.74% bio-jet fuel) and the lowest yield was observed when using 1% MB and 2% MB (33% and 33.76% bio-jet fuel) respectively. When no catalyst was used, a bio-jet fuel yield of 33.8% was obtained. The optimum yield (40%) was obtained using MB at 18 min and 4% catalyst.

#### Effect of reaction time

**Figure 7:**
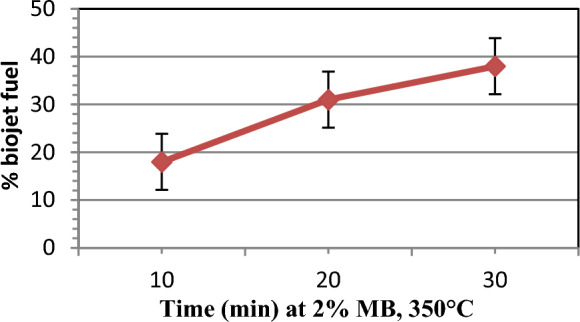
Time effect on yield of bio-jet fuel production.

Under the same conditions of 350 °C, 4 bar H_2_, MB catalyst was used in the hydro-catalytic cracking of Jatropha oil at different time (10–30 min) to produce bio-jet fuel, the specifications of which are shown in Tables 5, 6 and Figs. 7. It is seen from Fig. 7 that the reaction time has a pronounced effect on the product yield, especially in the bio-jet fuel cut. Three laboratory experiments were performed using 2% MB catalyst, at 350 °C, and for three different times 10, 20, and 30 min, it was observed that reaction pressure increased from 40 to 80 bar and the yield of bio-jet fuel increased during the process with the reaction time inside reactor. This is attributed to the completion of cracking reaction of long and heavy carbon chains to smaller chains to the produce the jet-fuel cut. The highest yield of 38% was at 30 min and the lowest yield of 18.3% was at 10 min.

### Bio-jet fuel specifications

#### GC/mass spectra analysis

**Figure 8:**
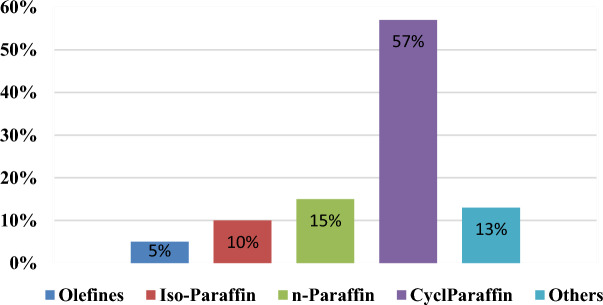
GC Mass % of different hydrocarbons in carbon length C8–C16 of bio-jet fuel.

In order to categorize the hydrocarbons contained in the product feedstock, the formation of alkanes, cycloalkanes, iso alkanes, and alkyl benzene during thermal catalytic cracking of jatropha oil was examined. The mixture of hydrocarbons contained n-paraffin, isoparaffin, cycloparaffin, olefins, and aromatics, according to the results. The typical optimum carbon length of C8-C16 in jet fuel is determined to be between 70 and 85%. According to Fig. 8, the total yield of paraffin content in the bio aviation fuel developed was close to 83%. The majority of iso and cyclo-paraffins, including methyldecane, alkyl cyclohexane, and iso octane (2,2,4-trimethylpentane), have one or two branched chains of alkanes, according to data from GC-mass spectra. This (57.01%) agrees with the literature ^42^.

The obtained unneeded olefins are interestingly close to the minimal (5% wt%) as in ideal jet fuel. The majority of iso, cyclo-paraffin has one or two branches as methyldecane and straight chain alkanes or branched chain cycloalkane as iso octane, according to data from GC/mass spectra.

In comparison to iso-octane (a branched alkane), molecules like methyldecane (an iso-paraffin) are substantially more prevalent in the petroleum jet fuel component. The most expensive and challenging to obtain component of jet fuel, iso-paraffin, was found in good concentration in the bio aviation fuel that had been manufactured. Because an increase in branching decreases the fuel's ignition point, the amount of branched alkanes has a significant impact on the purity of the fuel^43^.

#### Bio-jet blend engine test

**Figure 9:**
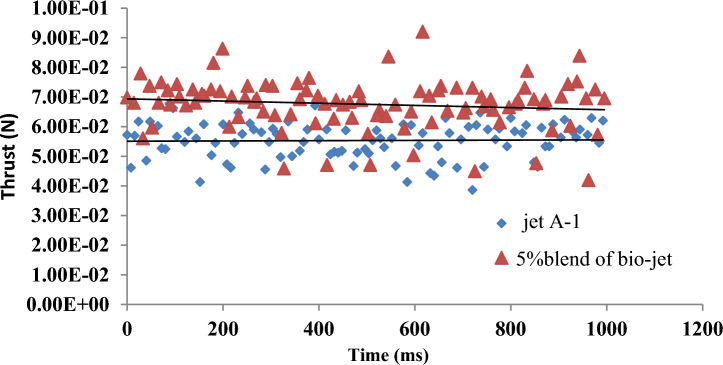
Thrust test results for 5% blend of bio-jet and jet A-1.

When the specifications of 5% bio-jet fuel produced from jatropha oil blend with jet A-1 were compared to industry standards, the results fell within the required range Table 7. The experiment showed that the thrust produced by utilizing 5% blend is reasonably consistent with jet A-1. According to Fig. 9 Red points show the different thrusts of the blend kerosene jet fuel and 5% bio-jet that were obtained in this work at various times during the testing experiment. This shows that the blend bio-jet has higher thrust values than pure petroleum kerosene. Blue points show the various thrusts of the petroleum jet fuel (kerosene jet fuel).

**Table 7:** Fuel specifications for the blend (5% bio-jet with 95% jet A-1).

Catalyst type	Density (g/ml)	Freezing point (°C)	Flash point (°C)	Existent gum mg/100 ml	Final boiling point (°C)	Sulphur, mercaptan, wt%
No–Catalyst	0.8241	−52	50	6.4	270	0
4% MB	0.8345	−56	53	5.9	270	0
Jet A-1^15^	0.775–0.84	−47 max	38 min	7 max	300 max	0.003 max

#### Fuel specifications for the blend 5% bio-jet

The density of the obtained bio-jet fuel after distillation is given in Table 7 and was in the range 0.8241–0.8345 g/ml at 15 °C. This conforms to the ASTM D1655 specifications of range of 0.775–0.84 g/ml.

Freezing point is one of the most important methods of testing aviation fuel products. As the plane ascends, temperatures in the fuel tank and lines can drop. If the temperature of the tanks and lines drops below the freezing point of the fuel, this will impede the flow of the fuel and close the filter. This will starve the engine and shut down. Table 7 also gives the freezing point values of the produced bio-jet fuel. It ranges between −52 °C and −56 °C and are comparable with the ASTM D2386 maximum value of −47 °C.

Transportation regulations distinguish fuel as flammable or combustible, depending on the point of ignition by measuring of fuel flash point. The produced bio-jet fuels flash point of 53 °C is well above the ASTM D2887 value of 38 °C.

The maximum gum content as specified by ASTM D-381 is 7 mg/100 ml, the high gum content can cause induction-system deposits and sticking of intake valves. Table 7 also presents the gum content of a blend of 5% of the produced bio-jet fuel with 95% jet A-1. The gum content is below the range specified by ASTM D-381. The results were obtained using the catalyst (MB) and without using a catalyst. Both of them produced the lowest gum content of 5.9 mg/100 ml and 6.4 mg/100 ml respectively.

## Conclusions

The acid modifications that applied to natural clay to generate catalytic activity are 0.1M HCl, heated to 110 °C, for 4h, calcination at 550 °C for 4h.

The catalyst (MB) was characterized physically and chemically by XRF, XRD, FTIR, and BET.

The catalyst was used in the production of bio-jet fuel by catalytic hydrocracking of Jatropha oil under different process conditions such as percentage variation of catalyst (1–4%) and reaction time (10, 20, and30 min), the obtained optimum operating conditions are 350 °C, 4 bar H_2_, 18 min reaction time, and 4% catalyst.

The biofuel mixture obtained from the reaction was separated according to their boiling point by distillation into four fractions: bio-gasoline (70–170 °C ), bio-jet (170–270 °C), biodiesel (270–330 °C), and mazot. The obtained bio-jet fuel yield was 40% from the total product.

The bio-jet fuel produced using the catalyst (MB) was tested and its specifications conform to the ASTM D1655 specifications with respect to freezing point (−56 °C), the flash point (53 °C), and existent gum content (5.9 mg/100 ml).

This result gave a promising potential in the bio-jet fuel manufacture from jatropha oil due to economic and environmental purposes, being of lower cost than vegetable oils and environmentally positive way for using blend of bio-jet fuel with jet A-1.

## Data Availability

The datasets used and/or analyzed during the current study available from the corresponding author on reasonable request.

## Abbreviations

H_2_: Hydrogen gas
B: The Egyptian Bentonite clay
MB: Modified Bentonite clay
ASTM: American Society for Testing and Materials
XRF: X-ray fluorescence
XRD: X-ray Powder Diffraction
FTIR: Fourier-transform infrared spectroscopy
BET: Brunauer, Emmett and Teller surface area
